# Risk of venous thromboembolism in relation to high physical activity level in men over 27 year follow up

**DOI:** 10.1007/s11239-024-03013-x

**Published:** 2024-07-09

**Authors:** P. Wändell, M. A. Enarsson, T. Feldreich, L. Lind, J. Ärnlöv, A. C. Carlsson

**Affiliations:** 1 Department of Neurobiology, Care Sciences and Society, Karolinska Institutet, Huddinge, Sweden; 2 School of Health and Welfare, Dalarna University, Falun, Sweden; 3 Department of Medical Sciences, Faculty of Medicine, Uppsala University, Uppsala, Sweden; 4 Academic Primary Health Care Centre, Stockholm Region, Stockholm, Sweden

**Keywords:** Physical activity, Venous thromboembolism, Cardiovascular risk factors, Strenuous exercise

## Abstract

Venous thromboembolism (VTE) is the third most common type of cardiovascular disease. An association between high level of physical activity (PA) and the onset of VTE has been found in some, but not all previous studies. We aim to study the association between PA-level and VTE in a cohort of men with updated data on PA levels at four occasions. We used data from the Uppsala Longitudinal Study of Adult Men (ULSAM) study initiated in 1970, a study of men at age 50 years (*n* = 2,294 at baseline) examined on leisure time PA by questionnaire and traditional cardiovascular risk factors. Examinations were repeated at ages 60, 70, and 77, and follow-up was completed after a median time of 33 years. Cox regression analysis with hazard ratios (HRs) using updated covariates for PA and risk factors was performed on the association of PA levels with incident VTE, with adjustments for established cardiovascular risk factors (systolic blood pressure, LDL- and HDL-cholesterol, BMI, diabetes, and smoking). Totally 186 men experienced a VTE during follow-up of 68,263 person-years at risk. Individuals with the highest PA level had an increased relative risk of VTE, adjusted HR, 2.22 (95% CI 1.05–4.67), when compared to individuals with the lowest level of PA. In this cohort of men with a follow-up of 27 years, the risk of VTE was increased at the highest PA level. Findings indicate that there could be an increased VTE risk with higher PA level including strenuous activities.

**Graphical Abstract:**

**Figure:**
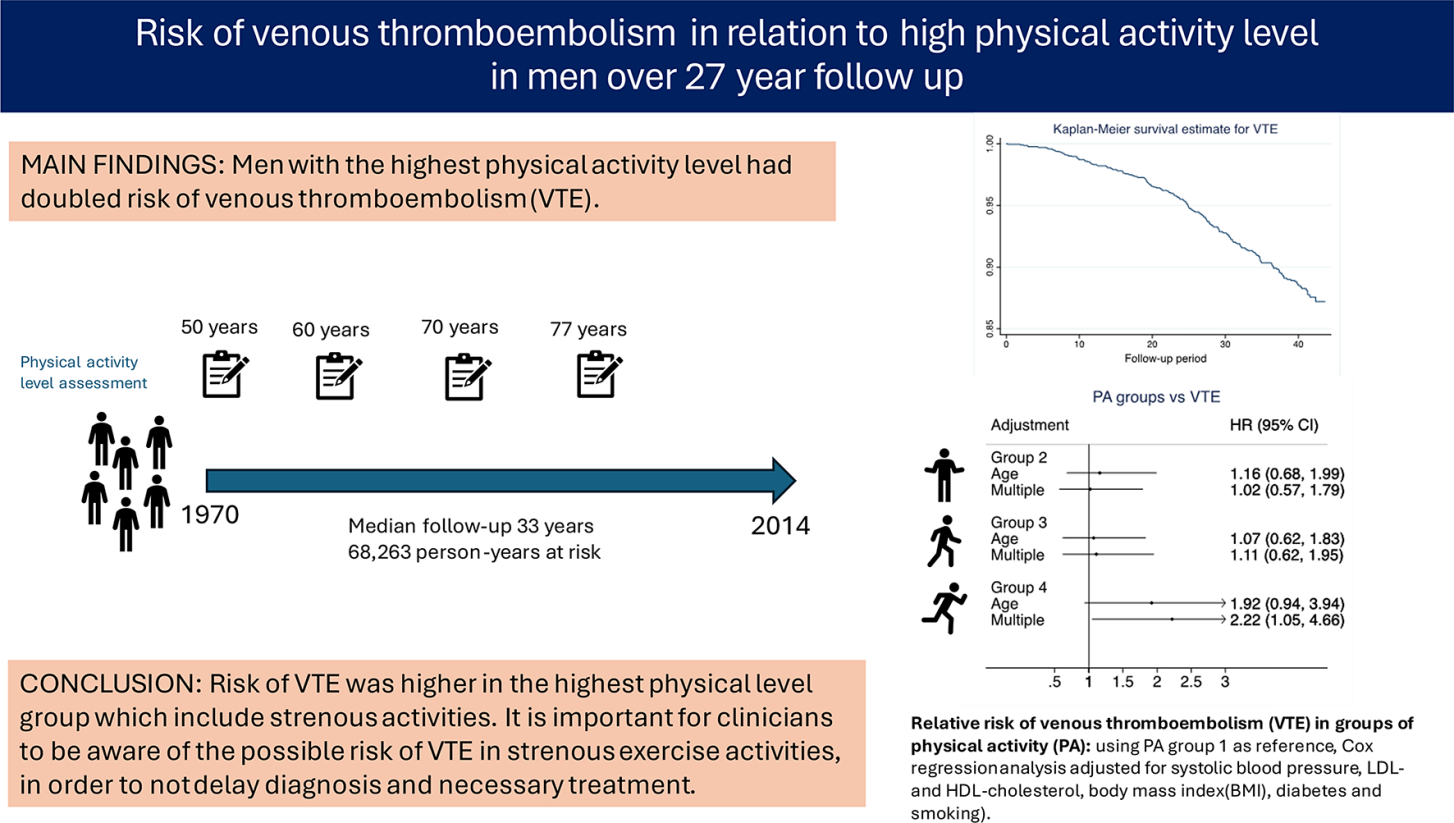
In this cohort of men with a follow-up of 27 years, the risk of venous thromboembolism was increased at the highest level of physical activity

## Introduction

Venous thromboembolism (VTE) is the third most common type of cardiovascular disease, after coronary artery disease and stroke [1]. In Sweden, the annual incidence of VTE has been estimated at 150 to 200 per 100,000 person-years, with an increased risk by age [2, 3].

The ways that VTE can express itself varies greatly, from asymptomatic thrombosis to fatal massive lung embolization. Anticoagulant treatment is the most effective way in preventing recurrence, but is associated with bleeding complications [4]. Models to predict recurrent VTE have been created, e.g. the Vienna model [5], showing a higher risk for men compared to women, patients with proximal deep venous thrombosis or pulmonary embolism, and higher levels of D-dimer.

Risk factors for cardiovascular diseases are well-known, but when looking at some risk factors such as hypertension, elevated cholesterol, diabetes, and cigarette smoking, these had no association with VTE [1]. Low Physical activity (PA) levels are also associated with a higher CVD risk [6], and mortality [7], including a higher risk of VTE which has been shown for bed rest and a sedentary lifestyle [8], in previous studies. This is in line with the results of two reviews concluded, that higher PA level showed lower VTE risk vs. low PA level [9, 10]. Thus, higher PA levels are generally associated with good health and well-being. A total of 150 min of PA of at least moderate intensity per week, or at least 75 min of high intensity PA weekly, is recommended by the Public Health Agency of Sweden, consistent with the recommendation from the World Health Organisation (WHO) [11].

However, there are also different health risks associated with PA especially strenuous exercise. The most documented disorders are different cardiovascular events including sudden cardiac death in long-distance running [12]. A Danish study found a U-shaped association between dose of jogging and all-cause mortality [13]. A previous review found that out of the 16 included studies, five found a U-shaped association between PA level and VTE risk, although non-significant in three of them [14]. As the studies defined PA levels in different ways there is a need for more studies, and especially studies investigating measurements of PA levels with individuals reporting strenuous activity.

The aim of this study was to explore the association between different levels of PA and incident VTE in a long-term follow-up of Swedish men that are reinvestigated with PA questions on four occasions.

## Methods

### Study samples

#### The Uppsala Longitudinal Study of Adult men (ULSAM)

The ULSAM study was initiated in 1970, and in 1970–74 2,322 men all aged 50 years living in the city of Uppsala, Sweden, were investigated as part of the Uppsala Longitudinal Study of Adult Men (ULSAM, http://www.pubcare.uu.se/ulsam) [15]. Of the invited men 82% accepted to participate. This cohort have since then been reinvestigated at ages 60, 70 and 77 years. All participants in ULSAM gave written informed consent, and the Ethics Committee of Uppsala University approved the study protocols. The study was conducted according to the Declaration of Helsinki.

### Traditional risk factors

The baseline examination of ULSAM in the early seventies when participants were 50 years old has been described in detail previously [6]. We included traditional cardiovascular (CV) risk factors, with LDL- and HDL-cholesterol, systolic blood pressure (SBP), BMI, diabetes, and smoking. Fasting blood samples were drawn in the morning after an overnight fast. Serum levels of cholesterol, triglycerides, and HDL were assayed by enzymatic techniques. Friedewald’s formula was used to calculate LDL-cholesterol. Moreover, fasting plasma glucose was measured using an oxidase method. Supine systolic and diastolic blood pressures were measured twice in the right arm after 10 min rest, and means were calculated. Data on smoking status at baseline was based on a questionnaire. BMI was calculated by weight/squared height.

### Physical activity

Leisure time physical activity was assessed by a self-reported questionnaire at each examination. Participants answering yes to the question best reflecting their activity level graded from 1 to 4: (1) Mainly sedentary behavior (reading, watching television, or activities which do not need physical activity). (2) Walking or cycling (for pleasure walking, cycling, or some other form of physical activity for at least 4 h per week). (3) Recreational sports or heavy gardening for at least 3 h every week (exercises to keep fit, heavy gardening, etc., for at least 4 h per week). (4) Regularly engage in hard physical training (hard training or participation in competitive sports, regularly and several times a week). The questionnaire categories has previously been validated and used by other studies [16, 17].

### Outcomes

VTE ICD-10 codes: I26, I80.2, I82.9; ICD-9 codes: 415.1 (415B), (416 W), (451X), (451B) (451 C), (453 W); ICD-8: 450, 452, 453. There was no loss of follow-up. The baseline examination was performed in 1970–1974 and data on cause-of-death and hospitalizations were obtained to December 31st, 2014, giving four decades of follow-up.

### Statistics

The analyses were conducted using Cox proportional hazard models using updated covariates for PA and risk factors at four occasions (50, 60, 70 and 77 years). The method of updated co-variates splits the time into intervals corresponding to the number of examinations. In this case the time is split into one 50 to 60 interval, one 60 to 70 interval, one 70-to-77-year interval and one interval from 77 years to censor date. Thus, each time interval is having its own “baseline” measurement of PA (and other covariates) and therefore it is only the actual level of PA that is the exposure during that time period, not the previous PA levels.

Time at risk was calculated from the date of examination until date of VTE end-point, date of death, or end of follow-up (31 December, 2014), whichever occurred first. PA was treated as an nominal variable with the sedentary group as referents and the other groups being compared to that referent. We added time-updated information on the traditional CV risk factors systolic blood pressure, LDL- and HDL-cholesterol, BMI, diabetes, and smoking to the models. We also investigated how much PA added to the discrimination of VTE obtained by the traditional CV risk factors by using logistic regression and area under the receiver operating curve (ROC), as an indication of the predictive value of PA, when added to a model with established cardiovascular risk factors.

## Results

Of the 2,322 men included in ULSAM, 2,294 had a valid recording of physical activity during a median follow-up of 33 years (maximum 44 years) at 50, 60, 70 and 77 years of age. During the follow-up period 186 individuals experienced a first-time VTE (3 were excluded owing to a VTE before baseline), with a total of 68,263 person-years at risk.

**Fig. 1:**
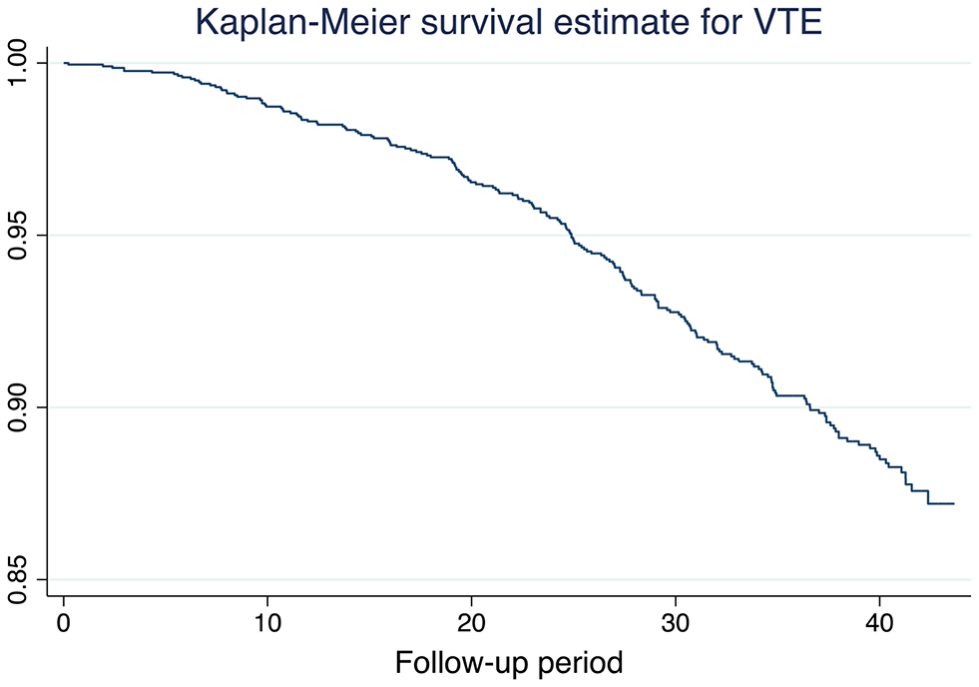
Kaplan-Meier curve for VTE during the follow-up period

The Kaplan-Meier survival curve for VTE over the follow-up period is given in Fig. 1. As expected, the number of events is sparse during the first years, but rather linear from the age of 70 years.

In Table 1 the mean values or percentages of the risk factors are shown.

**Table 1:** Basic characteristics (mean (SD) at the four examination cycles in the ULSAM-cohort for participants with full information on physical activity

	50 years (*n* = 2,175)	60 years (*n* = 1,616)	70 years (*n* = 1,092)	77 years (*n* = 762)
PA-level 1	11.4%	9.8%	3.9%	8.3%
PA-level 2	34.1%	52.0%	34.2%	35.4%
PA-level 3	48.5%	34.0%	55.4%	52.1%
PA-level 4	6.0%	4.1%	6.5%	4.2%
LDL-cholesterol (mmol/l)	5.2 (1.1)	4.4 (0.6)	3.8 (0.9)	3.4 (0.8)
HDL-cholesterol (mmol/l)	1.3 (0.3)	1.3 (0.2)	1.3 (0.3)	1.3 (0.3)
BMI (kg/m2)	24.9 (3.1)	25.4 (3.2)	26.1 (3.3)	26.2 (3.4)
Systolic blood pressure (mmHg)	133 (17)	142 (19)	146 (18)	150 (20)
Diabetes (%)	4	5	11	14
Smoking (%)	51	31	17	8

PA denotes physical activity LDL denotes LDL-cholesterol HDL denotes HDL-cholesterol SBP denotes systolic blood pressure

In Table 2, the results of the univariate analyses of the Cox regression, the PA groups and the cardiovascular risk factors are shown, with hazards ratios (HRs) and 95% confidence intervals (95% CI). There was a trend that the highest PA group showed a higher risk of VTE. In Table 3, the results of the multivariate cox regression are shown adjusted for established cardiovascular risk factors (systolic blood pressure, LDL- and HDL-cholesterol, BMI, diabetes, and smoking), with the highest PA group showing a significantly higher doubled risk.

**Table 2:** The relative risk of venous thromboembolic events (VTE) expressed as hazard ratios(HRs) with 95% confidence intervals (95% CI) with adjustments for age

	HR	Std error	z	*P* > z	95% CI
PA level 1	1 (ref)				
PA level 2	1.167	0.320	0.56	0.573	0.682–1.999
PA level 3	1.071	0.296	0.25	0.804	0.938–3.945
PA level 4	1.924	0.705	1.79	0.074	0.938–3.945
LDL (mmol/l)	0.887	0.068	1.57	0.117	0.763–1.031
HDL (mmol/l)	1.288	0.280	1.17	0.244	0.842–1.972
BMI (kg/m2)	1.036	0.024	1.54	0.124	0.990–1.083
SBP (mmHg)	0.999	0.004	0.22	0.828	0.992–1.007
Diabetes (%)	0.935	0.259	0.24	0.807	0.543–1.610
Smoking (%)	1.429	0.233	2.19	0.028	1.039–1.967

PA denotes physical activity LDL denotes LDL-cholesterol HDL denotes HDL-cholesterol SBP denotes systolic blood pressure

**Table 3:** The relative risk of venous thromboembolic events (VTE) expressed as hazard ratios (HRs) with 95% confidence intervals (95% CI) with adjustments for established cardiovascular risk factors

	HR	Std error	z	*P* > z	95% CI
PA level 1	1 (ref)				
PA level 2	1.017	0.295	0.06	0.953	0.576–1.897
PA level 3	1.110	0.321	0.36	0.719	0.629–1.957
PA level 4	2.224	0.841	2.11	0.035	1.059–4.668
LDL (mmol/l)	0.892	0.071	1.44	0.762	0.762–1.042
HDL (mmol/l)	1.301	0.294	1.16	0.245	0.835–2.025
BMI (kg/m2)	1.034	0.025	1.38	0.168	0.986–1.085
SBP (mmHg)	1.000	0.004	0.04	0.969	0.992–1.008
Diabetes (%)	0.092	0.275	0.28	0.781	0.513–1.652
Smoking (%)	1.439	0.252	2.08	0.038	1.021–2.027

PA denotes physical activity LDL denotes LDL-cholestero HDL denotes HDL-cholesterol SBP denotes systolic blood pressure

Figure 1 shows the associated risk of VTE in the PA groups. The higher risk in the highest PA group 4 is evident.

Table 4 shows the number of individuals and number of VTE events in each PA group at each period of the follow-up.

**Table 4:** Number of individuals and number of venous thromboembolism (VTE) events in each physical activity (PA) group at each examination period of the follow-up

PA-category	Examination period (age at years)	Nr of subjects	Nr of VTE events and %
PA1	50	324	7 (2.2)
PA2	50	800	20 (2.5)
PA3	50	967	21 (2.2)
PA4	50	111	4 (3.6)
PA1	60	172	5 (2.9)
PA2	60	856	34 (4.0)
PA3	60	528	16 (3.0)
PA4	60	62	3 (4.8)
PA1	70	43	2 (4.6)
PA2	70	370	9 (2.4)
PA3	70	619	18 (2.9)
PA4	70	66	3 (4.6)
PA1	77	62	2 (3.2)
PA2	77	271	17 (6.3)
PA3	77	399	21 (5.3)
PA4	77	34	4 (11.7)

**Fig. 2:**
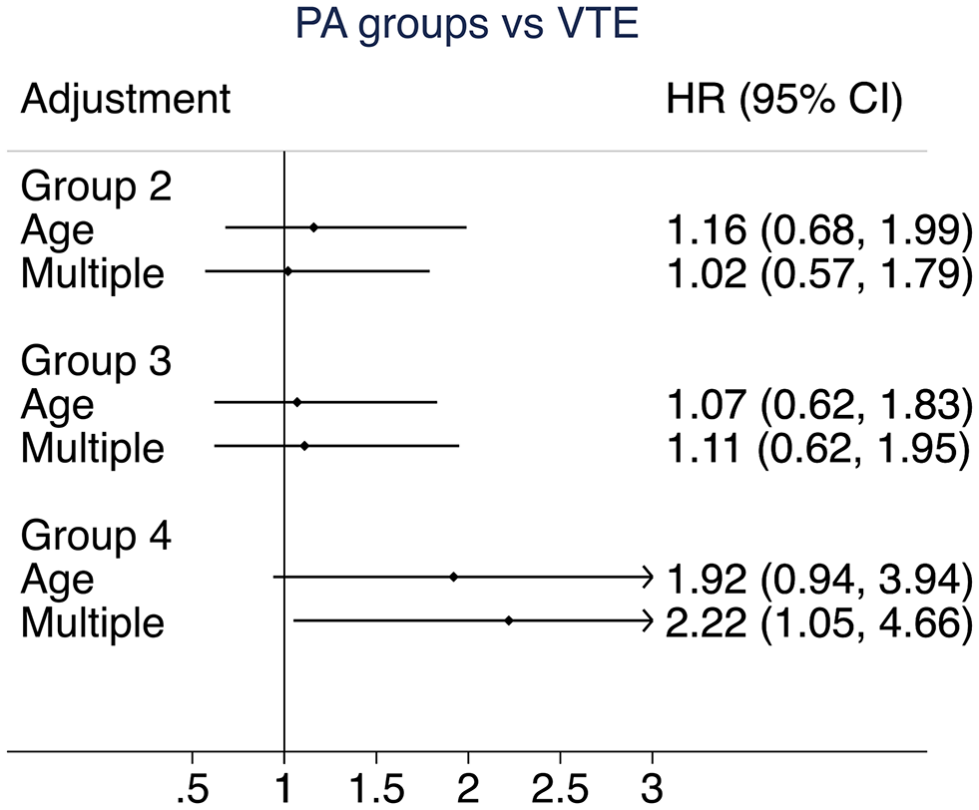
Risk of venous thromboembolism (VTE) in groups of physical activity (PA) using PA group 1, i.e., with the lowest leisure-time PA by the Cox regression

We also evaluated the prediction of VTE for the model with PA and the traditional CV risk factors, compared to the model with the traditional CV risk factors only without PA data, showing receiver operating curve (ROC) areas 0.591 vs. 0.589 (*p* = 0.89) See figure 2.

## Discussion

The main finding of this study was that the highest PA level showed a statistically significantly higher risk of VTE in models adjusted for established cardiovascular risk factors. This is in line with the studies having data on strenuous physical activity data in a previously published systematic review [14], which reported that in some studies, although not all, the highest PA level to be associated with an increased risk of VTE See Figure 2.

There are potential mechanism for the possible association between strenuous PA and incident VTE [18]. One suggestion according to earlier studies, also including review articles, is that exercise sessions including long and vigorous extreme exertion is associated with an imbalance between pro-thrombotic and fibrinolytic factors, thus causing hypercoagulability together with a weakened fibrinolysis [18–21], as the procoagulant parameters remain elevated longer than the fibrinolytic parameters, which return to baseline quickly. Furthermore, strenuous exercise seems to exert a more pronounced effect on platelet function among men, which could explain the higher VTE risk among them [20]. Another potential explanation is that the exercise may result in dehydration and that the high viscosity blood as a result may have a higher clotting probability [22, 23]. Third, strenuous exercise may cause microtrauma to the blood vessel walls, leading to endothelial injury, which could trigger clot formation [24]. Finally, aging is a known factor that increases the VTE risk [25], and the present study included participants included at the age of 50 years with a follow-up for 40 years.

Earlier studies have found some conflicting results, with only some of the included studies in the mentioned review finding a higher risk of high PA level [14]. This is not surprising, as PA was measured in different ways, and all measurements are not suitable to catch the more strenuous activities, which probably are responsible for the increased VTE risk.

Clinically it is important that exercise sessions including long and vigorous extreme exertion could be associated with VTE. The time interval between the strenuous PA session and the clinical diagnosis of a VTE could be rather long, why the association could not always be obvious. Besides, clinical signs of deep venous thrombosis could be misjudged in long distance runners and the diagnosis be delayed, as it is normal to feel exhaustion after exercise. More studies on the association between strenuous PA activities are needed, and PA needs to be assessed by more precise methods to capture this, when considering the most plausible explanation [18–21]. Knowledge of the association between strenuous PA activities are important both for health care providers and PA practitioners in order not to delay correct diagnoses of VTE.

There are some limitations of this study. The number of participants is fairly low, compared to other studies found in the earlier mentioned review. Moreover, only men with a high socioeconomic status are included and the prevalence of VTE is expected to be lower than the general population. This selected study population limits the generalizability of the results to the rest of the population. The crude assessment of PA by questionnaire gives broad groupings with risk of misclassification, possibly underestimating the results. However, the questionnaire categories should be regarded as reflecting common patterns of physical activity rather than a precise measure. The variables being used in this study are not generally predictive of VTE and there are likely other unaccounted for factors that could decrease the overall importance of physical activity on VTE risk. There may be other residual confounding factors that we could not adjust for in the present study, such as arthritis and chronic obstructive pulmonary disease, which will limit physical activity over time. Among the strengths are the high response rate, the careful examinations of the participants, and the long follow-up period in National Swedish registers of high quality with little loss to follow-up.

In conclusion, we found an increased risk of VTE with the highest PA level which include more strenuous activities. It is important that clinicians are aware of the possible risk of VTE in strenuous exercise activities, in order to not delay diagnosis and necessary treatment. It could also be good to alert participants in marathon races and other ultra competitions about the risks and early signs of VTE, so that they may seek care as soon as symptoms occur after strenuous exercise or long-distance races.

## Data Availability

The ULSAM study is listed in the SND (Swedish National Data Service). Access to data is limited, contact persons for ULSAM is Vilmantas Giedraitis (vilmantas.giedraitis@pubcare.uu.se).
